# Feeding and Disease

**Published:** 1903-11-07

**Authors:** 


### The Hospital.

A Journal of

The Medical Sciences and Hospital Administration.

Vol. XXXV.—No. 893. NOVEMBER 7, 1903.

### Feeding and Disease.

When we consider that the human race has been partaking of food, and gaining experience of its effects, for an unknown number of thousands of years, it is not a little curious to observe the differences of opinion which still exist with reference to the very first principles which should guide us in its employment. Physiologists have still much to learn with regard to the processes by which food is converted either into living tissue or into the force by which that tissue is enabled to fulfil its functions in the economy; but it might be expected that simple questions of quality and of quantity would long ago have received answers from which there could be no appeal. Such an expectation, however, is as yet far from being realised; and the general public may well feel unable to decide where doctors disagree. In the face of a steadily falling death-rate and of a marked diminution in the frequency of many serious forms of disease, we yet are told by a prominent politician that a large proportion, twelve millions if we remember rightly, of the inhabitants of these islands are on the verge of starvation; and we learn from a popular dramatist that if the "best" people would consent to dine once a day instead of several times, the alteration in their habits would be greatly to their advantage. So far, at least, the opinion of the ignorant laity seems to incline towards a recognition of the benefits to be derived from moderation, if not from actual abstinence; although, it must be confessed, their practice cannot be said to be in complete harmony with their principles. The "best" people, however that fraction of the human race may be defined, have certainly no monopoly of over-eating. If we turn for guidance to the medical profession, we find a remarkable diversity alike of doctrine and of practice. In the interesting debate to which we referred last week, on the uses of notification in pulmonary tuberculosis, several of the speakers referred as it were incidentally, and as a matter about which there could be no dispute, to the value of an ample dietary. Dr. Newsholme, in describing the benefit to be derived from even a month in a sanatorium, gave a prominent place to the effects of "abundant food." Dr. Sykes, of St. Pancras, placed "improvement of clothing and food" next to prevention of overcrowding as a remedial measure. Mrs. Scatherd referred to the deleterious effects of "patent foods," and so on; while there can be no question at all as to the popular belief in the value and efficacy of what is commonly called "nourishment," however little, in many circumstances, it may deserve the name, or however far that which is put into the stomach may be from effecting the desired entrance into the economy.

At the very same Congress, however, at which the ordinary, and, as they may almost be termed, the grandmotherly opinions about food and feeding thus found effective utterance, a paper was read by Dr. Rabagliati in which he attributed the prevalence of disease largely to over-feeding, and expressed his belief that an undue accumulation in the system of waste materials thence derived was mainly concerned in the production of liability to infection by affording conditions favourable to the vitality and the multiplication of pathogenetic bacteria. Dr. Rabagliati is a well-known and highly respected physician at Bradford, and the occasion referred to was by no means the first on which he has expressed the same opinion. He has published a book in which he sought to connect increase of cancer with over-feeding, or rather with the retention of waste products which he believes it to involve; and in which he not only especially condemned excessive consumption of starch, but selected Yorkshire pudding as an article of diet by which such an excess is frequently occasioned. He maintained that the chief object to be kept in view, with regard to the prevention of infective diseases, whether tuberculous or of other kinds, was the preservation of the immunity of the body, and that this was to be effected by strict moderation in eating. The best means to prevent fevers among people leading a town life would, he said, be attained by feeding children twice a day to the extent of about an ounce of food to each 10 lbs., or perhaps to each 8 lbs., of their body weight; and the same for adults from 21 to 50 years of age, after which one daily meal would be much safer. At the same time he laid great stress upon the risks attendant on the consumption of food of bad quality, such as diseased potatoes or diseased rice; and he by no means ignored the effects of contributory causes, such as bad air, in the production of the general result. We need hardly say that we hold no brief for Dr. Rabagliati, and we even think it probable that he underrates the power of the organism to cast off what is superfluous. There can be little doubt, however, that he has hit upon a real blot in our civilisation, and that food in excess of the bodily requirements is consumed by large numbers of people, or even, as a rule, by the whole community. We have spoken of "grandmotherly" views on the subject of the value of "nourishment," but we must not forget that our grandmothers were accustomed to lay stress upon two golden rules, namely, to eat slowly, and to rise from table with an appetite. The observance of the former gives the system time to recognise that its wants have been supplied; the observance of the latter would afford security against excess. Dr. Rabagliati has at least this argument in his favour, that the most remarkable examples of bodily and mental vigour prolonged to extreme old age have unquestionably been furnished by very moderate eaters; and his views as to the importance of quality will probably be confirmed by all competent observers. It is much to be wished that Parliament would take more thought than it has ever done hitherto with regard to the quality of provisions which are publicly sold, especially in poor localities, and that some authority would endeavour to bring home to the proprietors of certain patent foods the offence of endeavouring to obtain money under false pretences.

